# Effect of 1-Month Oral Oxycodone on Postoperative Pain Following Total Knee Arthroplasty

**DOI:** 10.2106/JBJS.OA.26.00170

**Published:** 2026-08-18

**Authors:** Mohammad Poursalehian, Seyed Hadi Kalantar, Mohammadreza Razzaghof, Mahboobeh Tajvidi, Sina Esmaeili, Maziar Nafisi, Abdolsalam Razzaghi, Mohammad Ali Mansournia, Hirad Farajidavar, Fatemeh Hashemipour, Mohammad Ayati Firoozabadi, Seyed Mohammad Javad Mortazavi

**Affiliations:** 1 Joint Reconstruction Research Center, Tehran University of Medical Sciences, Tehran, Iran; 2 Department of Orthopedic Surgery, Imam Khomeini Hospital Complex, Tehran University of Medical Sciences, Tehran, Iran; 3 Department of Epidemiology and Biostatistics, School of Public Health, Tehran University of Medical Sciences, Tehran, Iran

## Abstract

**Background:**

Opioid stewardship is a priority after total knee arthroplasty (TKA). Although scheduled, outpatient oxycodone adds clinically meaningful benefit to contemporary multimodal, nonopioid analgesia remains uncertain.

**Methods:**

We conducted a phase-II, single-center, triple-blind, randomized, placebo-controlled trial. Adults undergoing primary TKA received immediate-release oral oxycodone (5 mg twice daily) or matching placebo for 1 month in addition to a standardized multimodal regimen (acetaminophen, celecoxib, pregabalin). The primary outcome was knee pain on the Visual Analog Scale (VAS) on postoperative days 1, 7, and 30. Secondary outcomes were hospital length of stay, Knee Injury and Osteoarthritis Outcome Score (KOOS), Oxford Knee Score (OKS), Pittsburgh Sleep Quality Index (PSQI), patient satisfaction, and adverse events through 6 months. Analyses followed the intention-to-treat principle.

**Results:**

Between July 2023 and July 2024, 190 patients were randomized (95 per group); 2 placebo recipients died of myocardial infarction before the 6-month visit. Baseline characteristics were balanced. On postoperative day 1, the mean VAS pain was 6.7 ± 1.5 (mean ± SD) with oxycodone and 7.2 ± 1.5 with placebo (mean difference, 0.48 cm; 95% confidence intervals, 0.06-0.92; P = 0.015); the difference did not reach the minimal clinically important difference (MCID) (1.0 cm). No between-group differences were observed on day 7 or day 30. Length of stay was reduced by 0.2 days (1.7 vs. 1.9 days, P = 0.047), a statistically significant but clinically modest difference. KOOS, OKS, PSQI, and patient satisfaction were comparable at 1 or 6 months. Minor adverse events were similar between groups; no serious events were attributed to the study drug.

**Conclusions:**

In patients undergoing primary TKA with optimized multimodal, nonopioid analgesia, a fixed 1-month course of oral oxycodone produced small improvements in day 1 pain and length of hospital stay that were statistically significant but below established MCID thresholds. These findings support individualized prescribing through shared decision making rather than routine scheduled oxycodone use after TKA.

**Level of Evidence:**

Therapeutic Level I. See Instructions for Authors for a complete description of levels of evidence.

## Introduction

Knee osteoarthritis frequently necessitates total knee arthroplasty (TKA) for advanced joint degeneration^1^. Although TKA effectively improves function and quality of life, postoperative pain management remains challenging. Inadequate pain control can result in prolonged hospitalization or skilled nursing facility (SNF) placement, increasing infection risk; pain that hinders early mobilization may cause decreased range of motion, arthrofibrosis, and the need for manipulation under anesthesia or lysis of adhesions^2,3^.

Opioids are potent analgesics but carry significant risks including nausea, constipation, dependency, and diversion^4^. The opioid crisis has transformed prescribing practices, with stricter regulation and a strong shift toward multimodal nonopioid analgesia^5,6^. The arthroplasty community now favors opioid-sparing protocols incorporating acetaminophen, non-steroidal anti-inflammatory drugs, pregabalin, and regional techniques including adductor canal, genicular nerve, and interspace between the popliteal artery and capsule of the knee blocks^7-11^. Nevertheless, the role of scheduled outpatient opioids after TKA remains controversial: Some studies suggest modest benefits in pain relief and functional outcomes, while others highlight overprescription without clear clinical advantage^1,10,12,13^. Expert recommendations—not formal clinical practice guidelines—advocate limiting postoperative opioid prescriptions to 3 to 7 days; however, practice variation persists both within and between institutions^11,12^.

Despite these trends, the incremental benefit of scheduled outpatient oxycodone added to optimized multimodal analgesia after TKA remains uncertain. We conducted a phase-II, randomized, triple-blind, placebo-controlled trial to evaluate this question.

## Methods

### Trial Design and Oversight

This was a phase II, single-center, randomized, triple-blind, placebo-controlled, parallel-group trial conducted at Imam Khomeini Hospital Complex in Tehran, Iran. The trial was prospectively registered at the Iranian Registry of Clinical Trials (IRCT20220413054520N1) and approved by the Institutional Review Board; all patients provided written informed consent before randomization. The study received no external funding; the pharmaceutical provider supplied study drug and placebo but had no role in study conduct or analysis.

### Population

All patients with knee osteoarthritis who were undergoing primary TKA were eligible for this trial. Key exclusion criteria were use of antidepressant and antiepileptic medications, prior surgery on the affected knee, unwillingness to participate or follow-up, body mass index <20 or >40, regular alcohol consumption, opioid use within 3 months preoperatively (chosen to minimize confounding from baseline opioid tolerance; patients with remote opioid use >3 months prior for conditions unrelated to knee pathology were eligible), known hypersensitivity or contraindication to oxycodone or any other medications in the standard analgesic regimen, severe hepatic or renal impairment, active infection or inflammatory conditions affecting the knee joint, psychiatric conditions, pregnancy, lactation, and participation in other clinical trials

### Randomization and Blinding

Participants were randomly assigned 1:1 to immediate-release oral oxycodone hydrochloride (5 mg twice daily) or matching placebo for 1 month, using centralized web-based block randomization (Sealed Envelope). Allocation was concealed from patients, caregivers, investigators, and outcome assessors.

### Surgical Procedure and Anesthesia

All procedures were performed by 2 fellowship-trained orthopaedic surgeons using a standardized medial parapatellar approach with cemented posterior-stabilized implants. Patients received spinal anesthesia (10-15 mg of 0.5% hyperbaric bupivacaine at L3-L4 or L4-L5); general anesthesia was not used. Intraoperative periarticular infiltration analgesia included 300 mg ropivacaine, 30 mg ketorolac, 0.6 ml epinephrine (1:1,000), and 5 mg morphine in 100 ml saline, injected into key periarticular tissues^14^. No drains were used. Tranexamic acid (1.5 g) was given IV before tourniquet deflation. Dexamethasone was not administered as part of the perioperative protocol.

### Interventions and Postoperative Pain Management

All patients received standardized multimodal analgesia: celecoxib 200 mg twice daily, acetaminophen 500 mg every 6 hours, and pregabalin 75 mg twice daily from surgery through discharge; acetaminophen and celecoxib were continued as needed for 1 month postdischarge. The fixed twice-daily oxycodone schedule was chosen to preserve blinding; the total dose (5 mg twice daily; 15 morphine milligram equivalents [MME]/day; 450 MME total) is lower than some North American regimens (20-30 mg/day), which may have limited our ability to detect treatment effects. Adherence was high (>90% took ≥80% of doses).

For breakthrough pain, rescue analgesia consisted of intramuscular meperidine 25 to 50 mg every 4 to 6 hours as needed; no patient received intravenous morphine, hydromorphone, or patient-controlled analgesia. After discharge, patients reporting inadequate pain control could receive diclofenac suppository (50 mg every 12-24 hours for 3-5 days) or, rarely, additional opioids for severe refractory pain. Rescue medication use was not systematically tracked, representing a methodological limitation regarding total opioid consumption.

### Physical Therapy and Discharge

Physical therapy was initiated on the day of surgery following a standardized protocol (passive and active-assisted range-of-motion exercises, quadriceps setting, and assisted ambulation ≥15 meters); physical therapists were blinded to group allocation. Discharge criteria were standardized: (1) Visual Analog Scale (VAS) ≤5 at rest, (2) independent ambulation ≥15 meters with assistive device, (3) absence of surgical or medical complications, and (4) adequate home support. All patients were discharged directly to home on postoperative day 1 or 2; no patient was discharged same day or required SNF placement, reflecting institutional patient selection criteria and the family-supported home recovery context of our healthcare system.

### Rationale for Dosing Regimen

We selected a fixed-dose regimen of immediate-release oxycodone 5 mg twice daily (10 mg per day, 15 mg oral morphine equivalents) to preserve blinding, maintain stable plasma levels, and support opioid stewardship and regional prescribing norms. Although lower than typical North American regimens, this dosing minimized side effects and dependence risks but may have limited detection of treatment effects. Further study is needed to balance efficacy and safety across dose levels.

### End Points

All outcomes were collected via in-person interviews during clinic visits or, for patients missing appointments (9%), by telephone. Assessments were conducted by individuals blinded to treatment assignments.

The primary outcome was patients’ knee pain on days 1, 7, and 1 month after TKA, measured using the Visual Analog Scale (VAS, scores range from 0 to 10, with higher scores indicating greater pain intensity). VAS pain scores were assessed at standardized time points. Day 1 assessments were performed in late morning (10:00-11:00 am), approximately 3 to 4 hours after the morning study medication dose, coinciding with peak plasma levels of immediate-release oxycodone (time to maximum concentration 1-2 hours postadministration). Day 7 and day 30 assessments occurred during morning outpatient clinic visits (9:00 AM–12:00 pm), with patients instructed to take their morning study medication 2 hours before the appointment to again capture assessments near peak drug levels. Patients rated pain “right now” at assessment. We acknowledge that single time-point assessments do not capture the full 24-hour pain pattern or fluctuations related to activity and medication timing.

Secondary outcomes included hospital length of stay, knee pain and function, sleep quality, and patient satisfaction after TKA. Knee pain and function were evaluated with the Knee Injury and Osteoarthritis Outcome Score (KOOS) (42 items, 5 subscales; higher scores = better outcomes) and the Oxford Knee Score (OKS) (12 items; 0-48 scale, higher scores = better outcomes). Sleep quality was measured by the Pittsburgh Sleep Quality Index (PSQI) (0-21 scale; higher scores = worse sleep). Hospital length of stay, KOOS, OKS, and PSQI were assessed at 1 month, with KOOS and patient satisfaction also measured at 6 months. Adverse events were recorded throughout the 6-month follow-up via interviews, clinical visits, and medical record review.

### Statistical Analysis

The sample size was calculated based on a minimal clinically important difference (MCID) of 1.0 in the VAS for pain at 24 hours postoperatively, as identified in a previous systematic review^15^. The sample size calculation assumed a SD of 1.5 points for postoperative VAS pain, based on prior literature. Assuming a two-sided significance level of 0.05, 190 patients (95 per group) provided 90% power to detect this difference. Dropout was not considered since VAS was evaluated at 24 hours. MCIDs for secondary outcomes were 5.0 for OKS^16^, 7.9 for KOOS^17^, and 4.4 for PSQI^18^ at 1 month. With 190 patients, statistical power was 97% for OKS, 80% for KOOS, and 99% for PSQI. Continuous variables are mean ± SD; categorical variables are frequencies and percentages. Normality was checked via the Shapiro-Wilk test. Shapiro-Wilk testing demonstrated non-normal distributions for most continuous outcomes (P < 0.05), except overall KOOS scores at follow-up assessments. Because VAS pain was assessed at 3 prespecified postoperative time points (days 1, 7, and 30), multiplicity adjustment was performed using the Bonferroni method, yielding an adjusted significance threshold of P < 0.017 for the coprimary outcomes. Group comparisons used the independent-samples *t* test or Mann-Whitney *U* test, as appropriate. Treatment effects are reported as mean differences with 95% confidence intervals (CI). Analyses followed a prespecified plan, with missing outcome data assumed random. Missingness was assessed descriptively and via logistic regression on baseline covariates. Analyses were intention-to-treat using SPSS version 25.0 (IBM Corp., Armonk, NY). The detailed Statistical Analysis Plan is provided in Supplementary Appendix S1.

## Results

### Patients

**Fig. 1:**
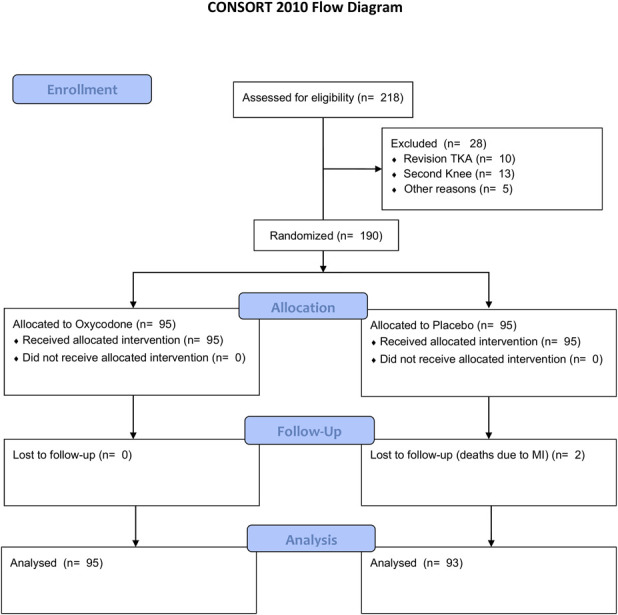
CONSORT flow diagram of patient enrollment, randomization, and follow-up.

Of 218 screened patients, 190 were randomized (95 per group); details of enrollment, allocation, and follow-up are shown in Fig. 1. Two placebo recipients died of myocardial infarction after discharge—at days 42 and 68, respectively—and were adjudicated as unrelated to study participation; both had multiple preexisting cardiovascular risk factors (Patient 1: 71-year-old man, hypertension and dyslipidemia, Revised Cardiac Risk Index [RCRI] = 1; acute anterior myocardial infarction [MI] with >75% left anterior descending occlusion. Patient 2: 68-year-old woman, hypertension, diabetes [HbA1c 7.2%], body mass index 32, RCRI = 1; acute MI. Neither patient had uncontrolled pain. Two deaths vs. none [P = 0.50] is consistent with published 90-day TKA mortality rates^19^). Baseline characteristics were balanced; 69% were female, mean age 65.1 ± 7.8 years (Table I). Because both deaths occurred after the day 30 assessment (at days 42 and 68), the complete-case and intention-to-treat analyses were identical for all 3 coprimary VAS outcomes. For 6-month secondary outcomes, a complete-case analysis (oxycodone n = 95; placebo n = 93) yielded results consistent with the intention-to-treat analysis, with no material differences in effect estimates or statistical significance across any outcome.

**TABLE I:** Characteristics of Participants at Baseline

Characteristics	Oxycodone (*N* = 95)	Placebo (*N* = 95)
Age—yr	65.4 ± 7.7	65.0 ± 7.9
Sex—no. (%)		
Female	62 (65)	69 (73)
Male	33 (35)	26 (27)
Height—meter	1.63 ± 0.09	1.63 ± 0.09
Weight—kg	76.6 ± 9.4	75.1 ± 9.5
BMI—kg/m2	29.1 ± 3.9	28.3 ± 3.9
Side of operation—no. (%)		
Right	47 (49)	53 (56)
Left	48 (51)	42 (44)
Baseline WOMAC	48.8 ± 8.4	50.4 ± 8.5
Baseline KSS knee score	44.6 ± 8.3	45.0 ± 7.5
Baseline KSS function score	40.4 ± 9.7	39.1 ± 7.6

BMI = body mass index, KSS = Knee Society Score, and WOMAC = Western Ontario and McMaster Universities Osteoarthritis Index.

### Primary Outcomes

**Fig. 2:**
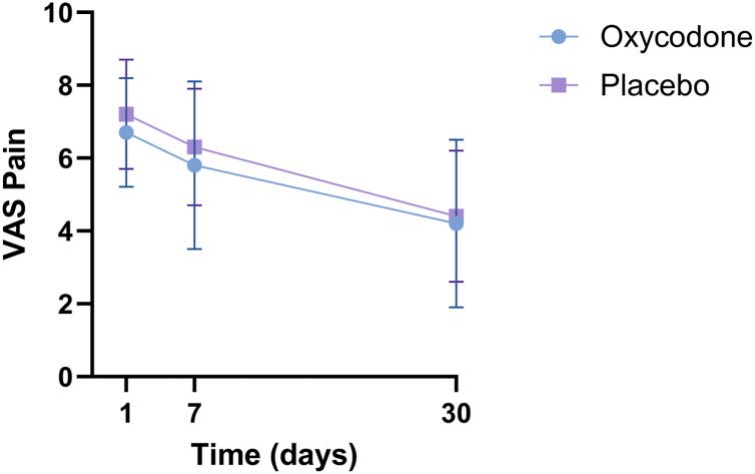
VAS pain scores after total knee arthroplasty. VAS = Visual Analog Scale.

On postoperative day 1, the mean VAS pain was lower in the oxycodone group (6.7 ± 1.5 vs. 7.2 ± 1.5; mean difference, 0.48 cm; 95% CI, 0.06-0.92; P = 0.015); however, the difference did not reach the MCID threshold of 1.0 cm. The day 1 finding remained statistically significant after Bonferroni adjustment for multiplicity (adjusted significance threshold P < 0.017). No significant differences were observed in VAS scores on postoperative days 7 or 30 (Fig. 2).

### Secondary Outcomes

The mean length of hospital stay (LOS) was shorter in the oxycodone group (1.7 ± 0.8 vs. 1.9 ± 0.8 days; mean difference 0.2 days, 95% CI 0.001-0.40; P = 0.047). This was statistically significant but of uncertain clinical importance (≈5 hours; no established MCID for LOS after TKA). In addition, discharge timing is influenced by multiple factors beyond pain control, including physical therapy progress, wound status, social factors, and bed availability. KOOS, OKS, PSQI, and patient satisfaction were comparable between groups at 1 and 6 months (Table II; see Appendix for complete outcome data).

**TABLE II:** Outcomes of the Trial

Outcome	Oxycodone (*N* = 95)	Placebo (*N* = 95)*	Mean Difference (95% CI)‡	P†
Primary outcome				
VAS Pain d 1	6.7 ± 1.5	7.2 ± 1.5	−0.48 (−0.92 to −0.06)	**0.015**†
VAS Pain d 7	5.8 ± 2.3	6.3 ± 1.6	−0.46 (−0.99 to 0.06)	0.085
VAS Pain d 30	4.2 ± 2.3	4.4 ± 1.8	−0.24 (−0.84 to 0.25)	0.300
Secondary outcomes				
Days of hospitalization	1.7 ± 0.8	1.9 ± 0.8	−0.20 (−0.40 to −0.001)	**0.047**†
KOOS symptoms at 1 mo	42.6 ± 18.9	43.4 ± 16.1	−0.80 (−5.85 to 4.25)	0.635
KOOS pain at 1 mo	28.7 ± 16.4	29.1 ± 16.1	−0.40 (−5.06 to 4.26)	0.551
KOOS ADL at 1 mo	30.3 ± 18.7	31.7 ± 17.2	−1.40 (−6.57 to 3.77)	0.576
KOOS recreation at 1 mo	14.5 ± 20.2	16.1 ± 20.8	−1.60 (−7.50 to 4.30)	0.385
KOOS QoL at 1 mo	18.8 ± 13.5	21.6 ± 16.6	−2.80 (−7.15 to 1.55)	0.508
Overall KOOS at 1 mo	27 ± 13.1	28.4 ± 13.3	−1.40 (−5.18 to 2.38)	0.545
OKS pain at 1 mo	20.7 ± 5.3	20.2 ± 5.1	0.50 (−1.01 to 2.01)	0.446
OKS function at 1 mo	14.5 ± 3.6	14.5 ± 3.4	0.00 (−1.01 to 1.01)	0.942
Overall OKS at 1 mo	35.3 ± 8.7	34.8 ± 8.2	0.50 (−1.94 to 2.94)	0.484
PSQI at 1 mo	8.2 ± 3.9	8.0 ± 3.9	0.20 (−0.91 to 1.31)	0.667
KOOS symptoms at 6 mo	74.2 ± 16.9	72.7 ± 19.9	1.50 (−3.84 to 6.84)	0.813
KOOS pain at 6 mo	71.1 ± 22.1	72.2 ± 22.8	−1.10 (−7.58 to 5.38)	0.624
KOOS ADL at 6 mo	66.8 ± 22.2	67.5 ± 23.9	−0.70 (−7.39 to 5.99)	0.778
KOOS recreation at 6 mo	37.2 ± 30.5	39.6 ± 31.8	−2.40 (−11.40 to 6.60)	0.608
KOOS QoL at 6 mo	45.4 ± 25.7	46.9 ± 27.9	−1.50 (−9.28 to 6.28)	0.777
Overall KOOS at 6 mo	58.9 ± 20.1	59.8 ± 22.2	−0.90 (−7.07 to 5.27)	0.788
VAS patients’ satisfaction at 6 mo	8.9 ± 1.2	8.8 ± 1.3	0.10 (−0.26 to 0.46)	0.849

All assessed outcomes were non-normally distributed (except for overall KOOS); therefore p-values are reported in nonparametric evaluation. Abbreviations and Score Interpretations: VAS = Visual Analog Scale for pain (range 0-10; 0 = no pain, 10 = worst imaginable pain; higher scores indicate worse pain; minimal clinically important difference [MCID] = 1.0). KOOS = Knee Injury and Osteoarthritis Outcome Score (all subscales range 0-100; higher scores indicate better outcomes): (1) KOOS Pain: 0 = extreme pain, 100 = no pain (MCID = 7.9), (2) KOOS Symptoms: 0 = extreme symptoms, 100 = no symptoms (MCID = 7.9), (3) KOOS ADL (Activities of Daily Living): 0 = extreme difficulty, 100 = no difficulty (MCID = 7.9), (4) KOOS Sport/Recreation: 0 = extreme difficulty, 100 = no difficulty (MCID = 7.9), (5) KOOS QOL (Knee-Related Quality of Life): 0 = worst quality of life, 100 = best quality of life (MCID = 7.9). OKS = Oxford Knee Score (range 0-48; 0 = worst outcome, 48 = best outcome; higher scores indicate better knee function and less pain; MCID = 5.0). PSQI = Pittsburgh Sleep Quality Index (range 0-21; 0 = excellent sleep quality, 21 = severe sleep disturbances; higher scores indicate worse sleep quality; MCID = 4.4). Patient satisfaction (range 0-10; 0 = completely dissatisfied, 10 = completely satisfied; higher scores indicate greater satisfaction). LOS = length of hospital stay (days).

Data are presented as mean ± SD unless otherwise indicated. P-values are from independent samples t-tests (for normally distributed continuous variables) or Mann-Whitney U tests (for non-normally distributed continuous variables).

* For outcomes that were assessed before 3 weeks all patients were included; for 1-month outcomes, 94 patients were included; for 6-month outcomes, 93 patients were included.

† Indicates statistical significance at p < 0.05.

‡ No between-group difference exceeded the predefined MCID thresholds.

### Safety outcome

Minor adverse events were similar between groups (oxycodone: n = 5; placebo: n = 4); nausea was most common. No serious adverse events were attributed to the study drug. The 2 placebo-group deaths (2.1%; 95% CI, 0.3%-7.4%) vs. none in the oxycodone group was not statistically significant (P = 0.50), consistent with published 90-day TKA mortality rates (0.3%-2.1%)^19^ (Table III).

**TABLE III:** Summary of All Adverse Events

Variable	Oxycodone (*N* = 95)	Placebo (*N* = 95)
Patients with no adverse events	90	89
Nausea	4	1
Flushing	0	1
Itching	1	1
Insomnia	0	1
Death	0	2

## Discussion

In this phase-II, triple-blind trial, adding a 1-month course of oral oxycodone (5 mg twice daily) to standardized multimodal nonopioid analgesia after primary TKA reduced pain only on postoperative day 1 and shortened LOS by approximately 5 hours—the results were statistically significant but fell below established MCID thresholds and were not sustained beyond day 1. No differences were detected in pain, function, sleep quality, or satisfaction at days 7, 30, or 6 months. These findings align with prior randomized controlled trials demonstrating that opioid-based regimens are not consistently superior to multimodal nonopioid protocols for postoperative pain control after TKA^1,20,21^. Opioids may not sufficiently control pain during movement, important for early rehabilitation^22-24^. In our trial, the multimodal regimen of acetaminophen, celecoxib, and pregabalin was generally effective, supporting opioid-sparing strategies^24^. Notably, dexamethasone was not administered in our perioperative protocol; its inclusion may further reduce early opioid requirements and warrants investigation in future trials. This article provides data relevant to international readers across diverse healthcare systems; however, generalizability is contextualized by our Iranian single-center setting, opioid-naive population, and absence of same-day discharge—all factors that differ from high-volume North American practice.

The LOS reduction (0.2 days; P = 0.047) requires cautious interpretation. No established MCID exists for LOS after TKA. Discharge was driven by multiple standardized criteria beyond pain control—physical therapy progress, wound status, and home support availability. Functional outcomes, sleep quality, and patient satisfaction showed no significant differences at 1 or 6 months, consistent with earlier studies^1,21^. Adverse events were mild and infrequent, with nausea most common and no serious opioid-related effects observed.^1,21,25^ All patients were discharged to home; none required SNF placement. The fixed twice-daily oxycodone schedule, adopted to preserve blinding, differs from pro re nata practice common in North America; a hybrid approach (scheduled for 3-5 days followed by pro re nata) might better balance analgesia and opioid minimization. Regional anesthetic techniques (peripheral nerve blocks, liposomal bupivacaine infiltration) substantially reduce pain in the first 24 to 72 hours—exactly the period where oxycodone benefit was observed. Future factorial design trials should determine whether optimized regional anesthesia renders scheduled oral opioids unnecessary^9,10^.

The restriction of benefit to day 1 has critical prescribing implications. TKA is associated with high rates of postoperative opioid prescribing; prolonged use may lead to opioid tolerance, dependence, and opioid-induced hyperalgesia^22,23^. Limiting opioid prescriptions at discharge reduces use without affecting pain control or recovery. Overprescription is common, with patients often taking far less than prescribed, highlighting unnecessary opioid exposure risks^26-28^. Opioid benefit appears confined to the immediate perioperative period, supporting expert consensus recommendations limiting postoperative prescriptions to 3 to 7 days^11,12^. Our 30-day regimen totaled 450 MME (5 mg twice daily × 1.5 conversion factor × 30 days), below the 600 to 900 MME threshold associated with increased risk of prolonged opioid use and opioid use disorder,^29,30^ which may partly explain the low adverse-event rate. Integration within enhanced recovery after surgery pathways—incorporating preoperative optimization, multimodal analgesia, early mobilization, and protocolized discharge—may further diminish the incremental value of scheduled opioids.^31-33^

Our data do not support routine, universal opioid prescribing after TKA with optimized multimodal analgesia; neither do they justify absolute prohibition. The modest early benefits observed must be weighed against well-established opioid risks, including dependency, diversion (illicit redistribution of prescription medications to unauthorized individuals), adverse effects, and contribution to the ongoing opioid crisis^4^. Based on our findings and existing literature^22,24^, responsible postoperative prescribing after TKA should include the following: (1) individualized assessment of pain expectations, prior experiences, and misuse risk factors (substance use disorder, benzodiazepine use, psychiatric comorbidities); (2) shared decision making with patients regarding opioid risks (constipation, sedation, falls, and dependence) and available alternatives; (3) lowest effective dose for the shortest duration, recognizing that most pain improves within 3 to 7 days; (4) as-needed rather than scheduled dosing to minimize total opioid exposure; and (5) robust nonopioid multimodal analgesia as the foundation, with opioids reserved for breakthrough pain. These principles, rather than defaulting to universal prescription or universal prohibition, best serve the individualized, patient-centered care that our results support^22,24^.

This study has several limitations. First, it was a single-center trial in an opioid-naive population undergoing primary TKA, limiting generalizability to patients with prior opioid exposure and to other cultural or healthcare contexts; multicenter studies are needed. Second, we did not quantify total rescue opioid consumption or assess psychological predictors of postoperative analgesic needs (e.g., catastrophizing, anxiety, depression), limiting subgroup analyses; future work should incorporate validated instruments and systematic analgesic tracking. Third, the conservative oxycodone regimen (5 mg twice daily) was lower than commonly used doses and may have reduced detectable efficacy, although it likely minimized adverse effects. Fourth, fixed scheduled dosing was required for blinding and may not reflect typical as-needed clinical practice. Fifth, the 6-month follow-up was insufficient to assess persistent opioid use or dependence beyond the 30-day treatment period. Sixth, regional anesthetic techniques were not used, which may limit applicability to contemporary TKA pathways where such strategies reduce opioid requirements. Finally, the sample size was powered for moderate VAS differences and may have been insufficient to detect smaller effects in secondary outcomes; the single-institution setting and standardized surgical pathway further limit external validity.

## Conclusion

In patients undergoing primary TKA with contemporary multimodal nonopioid analgesia, a fixed 30-day course of low-dose oral oxycodone produced a statistically significant but clinically modest reduction in day 1 pain and LOS, with no sustained benefit thereafter. For most patients receiving optimized multimodal analgesia, routine scheduled opioid prescribing after TKA provides minimal incremental clinical benefit beyond the immediate postoperative day. These findings support individualized, shared decision making over universal opioid prescribing. For patients who do receive postoperative opioids, regimens substantially shorter than 30 days, lower total doses, and as-needed rather than scheduled dosing may provide similar benefits while minimizing risk.

## Appendix

Supporting material provided by the authors is posted with the online version of this article as a data supplement at jbjs.org (https://links.lww.com/JBJSOA/B297). This content was not copy-edited or verified by JBJS.
